# Primary squamous cell carcinoma of the prostate: a case report of a rare clinical entity

**DOI:** 10.4155/fso.15.16

**Published:** 2015-11-01

**Authors:** Tithi Biswas, Tarun Podder, Pamela A Lepera, Paul Walker

**Affiliations:** 1 Department of Radiation Oncology, Seidman Cancer Center, Case Western Reserve University School of Medicine, Cleveland, OH 44106, USA; 2 Leo Jenkins Cancer Center, Brody School of Medicine, East Carolina University, Greenville, NC 27834, USA

**Keywords:** chemo-radiation, squamous cell carcinoma of prostate

## Abstract

Primary squamous cell carcinoma of the prostate is a unique and rare clinicopathological entity with fewer than 100 cases reported in the literature. Because of its rarity, the optimal management is not well known. Here, we report a case of primary squamous cell carcinoma of the prostate which was treated with definitive concurrent chemo-radiotherapy with excellent outcome along with a brief review of the literature.

## Abstract (lay abstract)

This is a case report about a rare form of prostate cancer called squamous cell cancer. It behaves more aggressively compared with more common types of prostate cancer. The treatment is controversial due to its rarity, but is different than that of the common variety of prostate cancer. From available limited information, it can be treated either with combined chemotherapy and radiotherapy or radical surgery for early stage disease.

**Figure 1.**
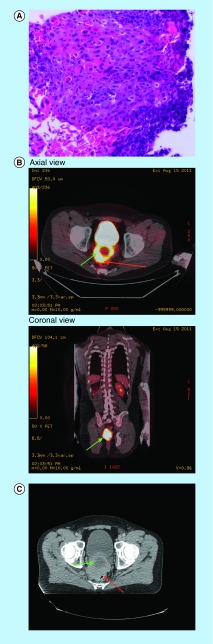
**Pre-treatment biopsy, and imaging studies.** **(A)** Initial biopsy from the rectal mass showing squamous cell carcinoma on H&E stain (high power microscopy). **(B)** (axial and coronal views) Initial staging positron emission tomography-CT scan showing hypermetabolic tumor involving prostate gland (green arrow) and the compressed rectum (red arrow). **(C)** Radiotherapy planning CT scan showing the abnormal looking prostate gland (green arrow) with rectum (red arrow) being pushed.

**Figure 2.**
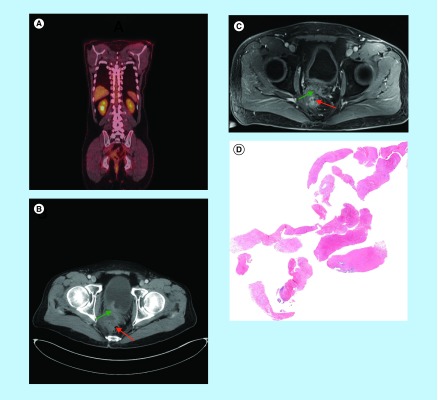
**Post-treatment imaging studies and biopsy result.** **(A)** Post-treatment positron emission tomography-CT scan showing complete metabolic response following completion of therapy. **(B&C)** Post-treatment CT and MRI scan showing excellent response (green arrow) to therapy with rectum in normal position (red arrow). **(D)** Post-treatment biopsy showing no malignant cells present (low power microscopy).

## Initial diagnosis

In June of 2011, the patient underwent laser transurethral resection of prostate for presumed benign prostatic hypertrophy. The tissue was not sent for histological examination. While his urinary symptoms were improved, he developed increasing anorectal discomfort along with lower back pain and bright red blood per rectum. He underwent an abdominal and pelvic CT scan on 8 July 2011, which showed enlarged prostate gland with indistinct borders merged imperceptibly with the rectum. The patient underwent a colonoscopy on 12 July 2011, which showed a large noncircumferential mass in the rectum extending from dentate line proximally to 13 cm. The rectal mass extends in the prostate with all layers of rectum being involved. A comment was made that the tumor has unusual appearance for a rectal tumor with almost normal appearing mucosa. The pathology from this mass was consistent with squamous cell carcinoma (SCC) (Figure 1A). An endoscopic ultrasound was performed which showed a hypo-echoic mass deep to the bowel, appeared originating from the prostate gland and extending into the wall of the rectum. His initial prostate-specific antigen (PSA) on 16 August 2011 at diagnosis was normal at 1.2 ng/ml.

A staging positron emission tomography PET- CT scan (Figure 1B – axial and coronal views) was performed, which revealed a hypermetabolic large prostate mass with likely invasion of the rectum. On the CT scan (Figure 1C), the rectum was clearly seen separate from the prostate tumor and being pushed by this mass. A regional hypermetabolic lymph node was also seen on the PET-CT scan. Therefore, he was staged as T4N1M0.

## Treatment

He was evaluated by the surgeon, and given the locally advanced nature of his malignancy, he was recommended neoadjuvant therapy followed by most likely pelvic exenteration. The patient underwent chemo-radiation concurrently. His chemotherapy regimen included Mitomycin C and 5-Fluoro-uracil (5FU) intravenous continuous infusion every 3 weeks for two cycles. He received pelvic radiotherapy with 45 Gray (Gy) in 25 fractions and an additional 9 Gy boost to the gross tumor to a total dose of 54 Gy.

## Outcome

Following completion of his therapy, he underwent a repeat PET-CT scan (Figure 2A), and CT scans (Figure 2B), MRI scans (Figure 2C), which showed complete metabolic response and no obvious tumor on Ct and MRI scans. He underwent examination under anaesthesia (EUA) and biopsy (Figure 2D). The pathology confirmed benign prostatic gland and no malignant tumor cells. On his last followup on 17 January 2014, which is 27 months later, the patient is doing well without any evidence of local or distant recurrence based on a repeated PET-CT scan.

## Discussion

Carcinoma of the prostate gland is an extremely rare malignancy accounting for less than 0.5–1% of all prostate carcinomas. The first case was described in 1926, and to date, about 77 cases have been reported including the current case [1]. The mean age at presentation is 59 years (range 52–79 years), [2] and our patient was 57 years old at the time of presentation. It is thought to be rather aggressive with an average survival of 14 months [1]. The pathogenesis of SCC is not fully understood. There have been case reports of secondary SCC of prostate following radiation treatment of more common adenocarcinoma of the prostate [3]. The cell of origin is also debatable. Some have thought the origin of this rare malignancy of prostate is the prostatic urethral mucosa, while others have concluded that it arises from the transitional epithelium of periurethral ducts or the basal cells of prostatic acini [4,5]. There is no specific immunohistochemical marker for this rare but aggressive malignancy. Lager *et al*. have postulated that SCC of prostate develops due to adverse stimuli affecting columnar cells and they lose their ability to secrete PSA and prostatic acid phosphatase but ability to produce keratin [6]. Inability to secrete PSA suggests most likely separate pathogenesis of this cancer and less likelihood to respond to standard androgen blockade that is done in prostate adenocarcinoma. Clinically, it may be indistinguishable from more common counter part adenocarcinoma of the prostate with presenting symptoms being urinary obstruction or pain secondary to bony metastasis, although the natural history is much different. It does not produce PSA. From the available limited case reports with complete information about treatment and outcome (Table 1), it appears that SCC of prostate can fail both locoregionally including pelvic lymph nodes but also distant sites including bones, liver and lungs. The bony lesions are usually osteolytic rather than osteoblastic seen in adenocarcinoma of prostate. Therefore, histological diagnosis is crucial with appropriate immunohistochemistry when appropriate to exclude more common adenocarcinoma subtype. In our patient, his initial transurethral resection of prostate tissue was not sent for histological diagnosis and the biopsy from his rectum showing squamous cell histology which initially was thought to be originating from anus. The subsequent imaging studies confirmed the primary site of origin to be prostate gland.

Because of the rarity of this malignancy, the treatment is controversial. From the limited case reports with complete information about treatment and outcome that are available in English literature, it appears that more aggressive therapy including surgery, or combined radiation and chemotherapy provides best outcome (Table 1) for organ confined disease. Because of distinguished pathogenesis, routine androgen blockade using orchiectomy should not be considered in palliative setting [7–13].

Of particular interest is the report by Munoz *et al*. [1]. They reported an encouraging result of treating with concurrent chemo-radiotherapy using cisplatin and 5FU, similar to the regimen used in SCC of anal origin. The patient remained disease free for 5 years when he relapsed locally and died. Similar to this, Majeed *et al*., Okada *et al*., Uchibayashi *et al*. and the current case showed greater than 15 months survival without any evidence of recurrence with combined chemo-radiotherapy. Our patient received similar chemotherapy regimen as anal cancer with excellent response. Few other reports with aggressive radical surgery with cysto-prostatectomy with or without chemotherapy suggested similar survival as well for organ confined disease.

Our patient was planned to undergo pelvic exenteration after completing neoadjuvant chemo-radiotherapy. However, his post-treatment PET-scan and MRI scan showed complete metabolic response and very nice radiographic response, respectively. In addition, he underwent examination under anaesthesia (EUA) with biopsy showing benign prostate tissue but no malignancy (Figure 2D)

This patient received concurrent chemo-radiotherapy utilizing Mitomycin C and 5FU iv. continuous infusion, the regimen commonly used in anal SCC. His radiation dose was much lower at 54 Gy compared with usual high dose of greater than 75 Gy, that is commonly used in adenocarcinoma of the prostate. With this regimen, he achieved complete metabolic and excellent radiographic response, that was confirmed with EUA and repeat biopsy, and he is still alive and disease free at 27 months. Even though he was originally planned for a pelvic exenteration following low dose radiation with chemotherapy which was subsequently avoided. Our result along with reports by Munoz *et al*., Mazeed *et al*., Okada *et al*. and Uchibayashi *et al*., of using concurrent chemo-radiotherapy approach with relatively low dose of radiation shows promise in this rare disease and may be employed instead of more radical surgery. Radical surgery may be used as a salvage therapy.

## Conclusion

Primary SCC is a rare but distinct clinico-pathological entity with rather aggressive natural course. From anecdotal case reports, it appears aggressive local therapy with concurrent chemo-radiation is a reasonable treatment option with encouraging survival for organ confined disease. Radical surgery can be utilized either as a primary approach or as a salvage therapy for local recurrence. The chemotherapy regimen commonly used and can be used, are the ones used for SCC of other anatomical sites like anal cancer or head and neck cancer.

## Future perspective

This is a rare cancer with limited knowledge. With improvement in our understanding, along with better imaging and better histological diagnosis, we will better recognize this entity as a complete separate disease from the common adenocarcinoma of the prostate. Even though it will be extremely unlikely that there will be a study comparing different treatment modalities, with many case reports, we will continue to refine the treatment recommendation.

**Table 1.** **Selected case reports with complete information about treatment and outcome that are available in the English literature.**

**Study**	**Age (years)**	**Sites of metastasis**	**Treatment; Surgery**	**Modality; Chemo-RT**	**Survival (months)**	**Ref.**
Mott IJ (two patients)	59, 65	Bone, osteolytic	Orchiectomy	DES, chemo, palliative XRT	5, 8	[7]
Sharma DP	69	Local, liver, lung	Pelvic exenteration, pelvic/inguinal lymphadenectomy		6	[8]
Majeed F	71	None	Radical prostatectomy	XRT, mitoxantrone, cisplatin	18+	[9]
Okada E	65	Iliac lymph nodes		XRT, PEP, cisplatin	18+	[10]
Uchibayashi T	72	None		XRT, bleomycin, cisplatin	21+	[11]
Imamura M	54	Local	Radical cystoprostatectomy	Methotrexate, peplomycin, cisplatin	60 +	[12]
Munoz F	76	Pelvic		cisplatin, 5FU, XRT	60	[1]
Sharma SK	65	None	Orchiectomy		0	[13]
Di Pietro C	72	Iliac lymph nodes, liver	TURP		0	[14]
Moskovitz B	65	Lung	Radical prostatectomy, TURP		5	[15]
Ulloa SA	83	Lung	TURP		13	[16]
Little NA (two patients)	56, 55	Pelvic lymph nodes and lung, none	Radical cystoprostatectomy		25, 40+	[17]
Nabi G (two patients)	60, 72	Osteolytic bone and lung and liver; bone and lung		Palliative XRT, adriamycin, methotrexate, folinic acid	4, 5	[2]
Gray G	65	Regional in perineum	TURP, APR		12	[18]
Corder MP	67	Nodes		XRT, chemo	5	[19]
Kanthan R (six patients)	42–85	Lungs, bone	TURP (3)	XRT (1), chemotherapy (4)	1–13	[20]
Malik RD	77	Pelvic Lymph nodes, lung		Pall. XRT	3	[5]
Present case	58	None	None	XRT, 5FU, mitomycin C	27+	-

5FU: 5-Fluoro-uracil; APR: Anterior pelvic resection; DES: Diethyl stilbestrol; PALL: Palliative; PEP: Peplomycin; TURP: transurethral resection of prostate; XRT: External bradiotherapy.

**Executive summary**

Primary *de novo* squamous cell carcinoma of the prostate is an extremely rare entity.
Only fewer than 100 case reports have been published in the literature.
Here, we report a case with primary squamous cell carcinoma of the prostate with brief discussion on review of literature.
